# Hospital Meetings, &c.

**Published:** 1899-01-28

**Authors:** 


### HOSPITAL MEETINGS, &C.

### METROPOLITAN HOSPITAL SUNDAY FUND.

The Earl of Stamford presided at a meeting of the Council of the Metropolitan Hospital Sunday Fund, held at the Mansion House on Thursday, 19th inst., for the purpose of electing the officers and committees for the year. A communication was made by Mr. C. Cheston, who acts as honorary solicitor to the Fund, to the effect that as a result of his inquiries he found that the residuary estate of the late Mr. Vokins, bequeathed to the Fund subject to the life interest in it of that gentleman's wife, would amount to about £49,000 Consols, worth to-day about £55,000.

Sir Edmund Currie expressed the indebtedness of the Council to Mr. Cheston for the time he had given and the trouble he had taken in connection with this munificent bequest, and a cordial vote of thanks was passed to Mr. Cheston for his services.

The re-election of the Committee of Distribution, the name of the Right Hon. William Lidderdale being substituted for that of Sir Stuart Knill, deceased, was agreed to, as was also that of the General Purposes Committee. The arrangement by which the secretary, with any two members of the Council, may act as the committee for surgical appliance orders and hospital letters, and which has been found so satisfactory in the past, was again adopted. The hon. secretaries, Sir Edmund Hay Currie and Mr. Richard B. Martin, and the secretary, Mr. Custance, who have held their offices for twenty-seven years—since the starting of the Fund—were reappointed, as were also Messrs. W. H. Pannell and Co., the honorary auditors.

This being the first Council of the year, Sir Henry Burdett thought it desirable to make the suggestion that, since the Fund had not been doing quite so well as in some former years, it was well worth the attention of the Executive Committee to consider whether steps could not be taken to draw public attention to the work. If this were not done, such institutions were apt to get out of sight and out of mind with large sections of the community. He would suggest that information be supplied to the Press—original facts and statistics of which they could make use. The editors were willing to help if they were helped to do so, but it had not been the practice of late years to approach them with copy of journalistic interest, and he certainly thought this might be done with advantage, say in the week immediately preceding Hospital Sunday. It was a matter of experience that the interest in any subject, whether political or social, was likely to flag if they did not keep pegging away. What was wanted was to arouse the attention of those who went and of those who did not go to the churches. The clergy wanted the sympathy and the support of public opinion behind them, and the encouragement of the lay public. It had been said that their Secretary did not interest himself sufficiently in hospital politics. He (Sir Henry) considered that the strength of Mr. Custance lay in his impartiality and his isolation, but it was certainly desirable that they should be somewhat more active and aggressive in their policy, and that a move should be made in the direction of arousing public opinion and interest through the newspapers. He therefore moved that the General Purposes Committee be requested to consider if any and what steps could be taken with a view of bringing the Hospital Fund more prominently before the notice of the public.

The Rev. C. H. GRUNDY seconded the recommendation, and also threw out the suggestion that it would be a good thing if, about a fortnight or so before Hospital Sunday, the Lord Mayor were to issue an invitation to the clergy of all denominations—and perhaps their wives—to a conference at the Mansion House to talk the matter over and give each other hints and suggestions. He also thought the *Lancet* and THE HOSPITAL might suggest suitable texts, and even give sketches of sermons that would be appropriate and effective, as a help to busy clerics.

Mr. WAKLEY referred to the fact that 125,000 copies of the *Lancet* were sent out containing the results of the independent inquiry made each year by that paper. One of these, he said, was sent to the editor of every paper, who was thereby supplied with facts and figures.

Sir HENRY BURDETT, while acknowledging the usefulness of the *Lancet's* efforts, observed that editors, after all, were but human, and that being so they liked to have special information supplied them. He reminded the Council that in the year when the largest sum the Fund had collected was raised he himself had arranged that it should be advertised in the press, and this had undoubtedly brought in a considerable sum. He deprecated managing such a Fund on too economical lines.

Sir EDMUND CURRIE expressed approval of the idea, and mentioned that Sir Sydney Waterlow, before he went abroad, had urged the adoption of such a proposal as that of Sir Henry Burdett—than whom, he declared, the Fund and hospitals generally had no better friend.

The proposition was put to the meeting and unanimously agreed to.

Dr. GLOVER proposed the election of Dr. Stephen McKenzie to the Council, in the place of the late Dr. Hare.

Sir HENRY BURDETT, in seconding this, said he knew that Dr. McKenzie took great interest in the Fund. If elected he would be a regular attendant at the meetings and his assistance would be of great service to the Council.

The motion was agreed to *nem. con.*

The only remaining business was the question of the election of someone in the room of Canon Eyton. Canon Fleming was expected to propose the name of Mr. Pennefather; but in his absence, and after some remarks by Sir Edmund Currie and others, and the statement by the Secretary that Canon Eyton's resignation had not yet been received, it was agreed, on the suggestion of Sir Henry Burdett, to let the matter stand over.

A vote of thanks to the chairman brought the proceedings to a close.

### DEACONNESS' INSTITUTION AND HOSPITAL.

A WELL-ATTENDED public meeting was held at the Public Hall, Forster Place, Tottenham, on Tuesday evening, the 24th inst., under the presidency of Sir HENRY BURDETT, K.C.B., to call attention to the new scheme recently sanctioned by the Charity Commissioners altering the constitution of the hospital, and to bring home to the inhabitants of Tottenham and its neighbourhood the need the hospital stands in of local financial assistance.

After letters expressing sympathy with the objects of the meeting had been read, amongst them being one from Mr. Samuel Morley, on behalf of the descendants of Mr. John Morley, who, with Dr. Laseron, founded the hospital,

Mr. JOSEPH HOWARD, M.P., moved the following resolution :—

"That this meeting hails with satisfaction the provisions of the new scheme, which by means of a popularly elected committee of management secures to the Tottenham Hospital a large measure of public control, and being of opinion that the hospital is deserving of the support of all the inhabitants of the district, hereby resolves to use every effort to raise the funds necessary to place it upon a sound financial basis."

He said that what the institution really wanted now was a large measure of public support and a large number of annual subscribers. It was only by getting these that they could hope to see this hospital a financial success.

Dr. T. GILBART SMITH, in seconding, said that there had no doubt been differences and difficulties in connection with the Tottenham Hospital in the past, owing to the fact that there had been no one at the tiller when the ship was in stormy waters; but now, under the new scheme, there was a good helmsman provided, in the shape of a committee which safeguarded all parties concerned. Not only would the sick be thought of and cared for, not only would the rights of the medical staff be considered and safeguarded, but the rights and wishes of the original founders of the institution would be conserved. Under this scheme the people of Tottenham not only had a good hospital at their doors, but they could have it managed by themselves and the sick cared for and looked after by local Tottenham men.

Sir HENRY BURDETT, speaking in support of the resolution, said he must tell them frankly that when he first looked closely into the condition of this institution and when he discovered that they had, on the one hand, an income of some £1,250 at the outside, and on the other an expenditure of something like £5,000, and that the institution had no endowment whatever, he felt the position to be very grave indeed. While thinking the matter out, however, he had recollected what had been done in another district of North London—Highbury; how the inhabitants of that neighbourhood had raised something like £100,000, had built a hospital (which, in his judgment, was one of the completest so far as its sanitary, hygiene, and administrative arrangements were concerned), had given it an income of something like £10,000, and had put by an invested fund of something like £30,000. Remembering this, remembering that Tottenham was some five or six miles from that other hospital, that there was within a radius of five miles a population of at least 200,000 to 250,000, and remembering what had been done in provincial cities with something like 60,000 or 70,000 inhabitants, he had come to the conclusion that now the inhabitants had the opportunity of having a hospital of their own, administered by their own people, and attended by their own local doctors, the money would be forthcoming, because it was absolutely needed by the poor residents of the district. He had no doubt that in five years' time, inspired by the success of their neighbours a little nearer London, they would have braced themselves up and proved that Tottenham men and women were as generous as their neighbours, and perhaps even a little better. (Loud applause.) The number of beds to population in the Northern District of London was only about one to

3,000 inhabitants, a very bad state of affairs, when it was realised that the minimum number of beds generally considered necessary was one to every 1,000 inhabitants. In conclusion, Sir HENRY said he had no doubt in his mind that the people of the district would soon enter into the possession, not only of the buildings of the Tottenham Hospital, but of the buildings fully equipped and fully occupied by the poor sick and suffering of the district.

Dr. HOOPER MAY, who had worked in the hospital for over 30 years; Mr. HERBERT NEILD; Mr. WILKINSON, a member of the old council of the hospital for over 27 years; and Dr. WARD, representing the medical practitioners of the district who were not on the staff, also supported the resolution, which was carried unanimously.

A most cordial vote of thanks to the chairman, proposed by Mr. J. PEDLEY, seconded by Mr. J. COHEN, brought a very successful meeting to a close.

